# Role of *miR153* and *miR455-5p* Expression in Oral Squamous Cell Carcinoma Isolated from Plasma

**DOI:** 10.31557/APJCP.2021.22.1.157

**Published:** 2021-01

**Authors:** Sajjad Baber, Mohammad Bayat, Abdolreza Mohamadnia, Ahmedreza Shamshiri, Pooyan Amini Shakib, Naghmeh Bahrami

**Affiliations:** 1 *Department of Oral and Maxillofacial Surgery, School of Dentistry, International Campus, Tehran University of Medical Sciences, Tehran, Iran. *; 2 *Craniomaxillofacial Research Center, Shariati Hospital, Tehran University of Medical Sciences, Tehran, Iran. *; 3 *Chronic Respiratory Diseases Research Center, National Research Institute of Tuberculosis and Lung Diseases (NRITLD), Shahid Beheshti University of Medical Sciences, Tehran, Iran. *; 4 *Department of Biotechnology, School of Advanced Technologies in Medicine, Shahid Beheshti University of Medical Sciences, Tehran, Iran. *; 5 *Research Center for Caries Prevention, Dentistry Research Institute, Department of Community Oral Health, School of Dentistry, Tehran University of Medical Sciences, Tehran, Iran. *; 6 *Department of pathology School of Dentistry, Tehran University of Medical Sciences, Tehran, Iran. *; 7 *Department of Tissue Engineering and Applied Cell Sciences, School of Advanced Technologies in Medicine, Tehran University of Medical Sciences, Tehran, Iran. *

**Keywords:** Oral squamous cell carcinoma, miR 153, miR455-5p, Real time Polymerase chain reaction

## Abstract

**Background:**

Despite the notable advances in modern surgery and radiotherapy,no significant increase in the five year survival rate of oral squamous cell carcinoma has been reported. Collecting evidence demonstrates that *miR 153 *and *miR 455-5p* play a key role in growth and progression of oral cancer. Early detection of OSCC is important for enhancing patient quality of life and clinical treatment.For this reason, biomarkers or tumour markers offer an opportunity to intervene and avoid development of oral cancer.

**Methods:**

A total of 50 blood samples from patients from both genders (25 OSCC and 25 healthy people/control groups) were obtained to determine the expression of *miR153* and *miR455-5p* using Real time Polymerase chain reaction and t test.

**Results:**

In general by using the formula Δ ct, it is evident that the *miR 153* expression in peripheral blood is lower in patients than in healthy individuals (1.97) while the *miR 455-5p* expression in peripheral blood is higher in patients than in healthy individuals (2.56).

**Conclusion:**

We conclude that *miR153* and *miR 455-5p* expression in serum can function as a diagnostic screening test for the early detection of oral squamous cell carcinoma.

## Introduction

Oral squamous cell carcinoma is a malignant tumor which is derived from stratified squamous epithelium (Tumuluri et al., 2002). It is one of the prominent cancers in the oral cavity (Ramos et al., 2010). According to cancer today, OSCC is also the sixteenth common malignancy for about 2% of all cancers in the world (Ferlay et al., 2018). About 90% of cancer deaths occur due to metastases (Guan, 2015). The prevalence of oral squamous cell carcinoma is higher in males as compare to females (Johnson et al., 2011). Common etiology is tobacco, alcohol and betel nut chewing.Viruses such as human papilloma virus, nutritional deficiency such as niacin deficiency and various antioxidants, advance aging and radiation i.e. ultraviolet and ionizing radiation with chemical exposure can lead to formation of SCC (Mehrotra and Yadav, 2006). Now a day, the incidence of oral cancer is high in younger generation due to heavy use of tobacco related products. It is more alarming that the prognosis of OSCC in younger generation is poor in comparison to middle age or older individuals up to 70 years of age (Vargas-Ferreira et al., 2012).

Given the recent decrease in cancer incidence rates, long-term mortality rates remain constant. Early stage screening is one of the most important elements for overhauled cancer survival. Biomarkers or tumour markers offer an opportunity to intervene and avoid development of cancer. Tumour markers are substances released into the blood by cancer cells (Sharma, 2009).Bence jones first described the tumour marker in 1846. He incidentally detected a heat precipitate in urine samples of patients suffering from multiple myeloma (Schrohl et al., 2003). Tumor markers are used to ascertain how well a patient responded to treatment and to search for recurrence of tumors (Sharma, 2009).

OSCC is mainly associated with a variety of micro RNAs i.e. a class of gene regulators. MicroRNA [miRNA] is repetitive and non encoding short sequences of 18 to 22 or 24 nucleotides of the RNA genus (Mendell, 2005; Garzon et al., 2009). It was initially discovered in round worm by Victor Ambros laboratory in 1993 (Ambros and Ruvkun, 2018). More than 60% of proteins encoding genes are controlled by the action of micro RNAs (Catalanotto et al., 2016).

Due to remarkable similarity of OSCC to inflammatory lesions and long term asymptomatic ability, two-thirds of patients are typically diagnosed with advanced stages of stage three and stage four cancers.Therefore early diagnosis will help in reducing the complications of OSCC (Mücke et al., 2009; Sklenicka et al., 2010).

Since 2008, circulating miRNAs are known as diagnostic biomarkers and important approaches for the blood based identification of human cancer. Screening for novel diagnostic biomarkers seems to be a dire need in dealing with OSCC patients (Mitchell et al., 2008).

*miR 455-5p* has inhibitory role in OSCC and the promising therapeutic goal of its treatment. It was found that *miR-455-5p* expression is regulated by the TGF-β-dependent pathway, which subsequently leads to UBE2B [Ubiquitin-conjugating enzyme E2B] down-regulation and contributes to oral cancer tumourigenesis (Cheng et al., 2016).

*miR 153* is significantly related to metastasis and poor prognosis in oral cancer. Expression of* miR* in tumour cells could inhibit TGF-β induced EMT and regress mesenchymal like cells to an epithelial trait. Hence miR 153 decreased expression amounted to an EMT incursion of tumour cells (Xu et al., 2013)

The aim of this study is early diagnosis of OSCC with the help of tumor markers i.e. *miR153* and *miR455-5*.

## Materials and Methods

We started our research on 50 patients in which 25 patients are diagnosed with primary SCC. 25 patients were selected as healthy subjects in control group. Their age and gender distribution are presented in Table 1.

Blood samples are collected in a vacutainer tube or clot activator tube after properly obtaining informed consent from patients with Oral SCC and healthy subjects.

*Inclusion criteria*

People with age 25 to 75 year, irrespective of gender are included in this study. OSCC may grow from any epithelial dysplasia. That is why preexisting lesions such as leukoplakia or erythroplakia are included in this study.

*Exclusion criteria*

This study will exclude people who have a history of undergoing treatment including chemotherapy, radiotherapy and previous OSCC surgery. Any other malignancy or diseases associated with OSCC are excluded in this study.

After about 60 minutes, we separate the serum from the tube by the process of centrifugationat 2,300 to 2,500 rpm for about 15 minutes.The total RNA was extracted including miRNA from a minimum of 200µm of sample by using the miRN easy serum/plasma advanced kit (Qiagen)which has outstanding results without the involvement of chloroform or phenols.

Real time PCR was performed using a SYBR Green PCR Master Mix with a brand name, Pars Genom Iran on real-time PCR instrument. We used the cDNA synthesis kit for production of c DNA from the extracted RNA of about 2µg. In real time RT-PCR, the produced cDNAs were used. Reverse transcriptase reactions involves: 2µg RNA sample, 0.5 mmol/L each of dNTP, 2×RT buffer, 50nmol/L stem-loop RT primer and 4 U/µLM-MLV reverse transcriptase. After that, reactions were processed in a PCR system at 37-38°C for 45-50 minutes and 85-87°C for 4-5 minutes.The specimens were then stored at 3-4°C.

We did our experiments in a concentration of approximately ,1µL universal primer, 25µL containing 60ng cDNA and 15 pmol each primers with 10µL 2× SYBR Green PCR Master mix. The PCR amplification reaction consists of denaturation at 95-97°C for 5 seconds followed by 40 cycles at 62-65°C for 20 seconds and 72-74°C for 30 seconds.We analyzed the variations in expression level in each category.The whole procedure was repeated three times in order to find a median value.

*Statistical methods*

We performed our statistical analysis by using SPSS 26 software. We summarize quantitative variables as mean and standard deviation (considering normal distribution) and categorical variables as count and percentage. For comparing gene expression between study groups t test was performed. For checking interaction of gene expression by gender or age groups GLM (generalized linear model) was used. Level of statistical significance was set to 0.05.

## Results

**Figure 1:**
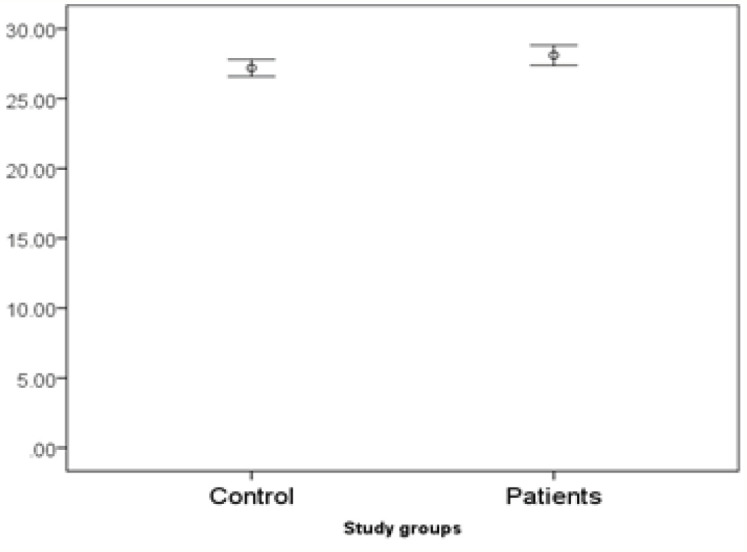
Expression of miR455-5p in Study Groups

**Figure 2:**
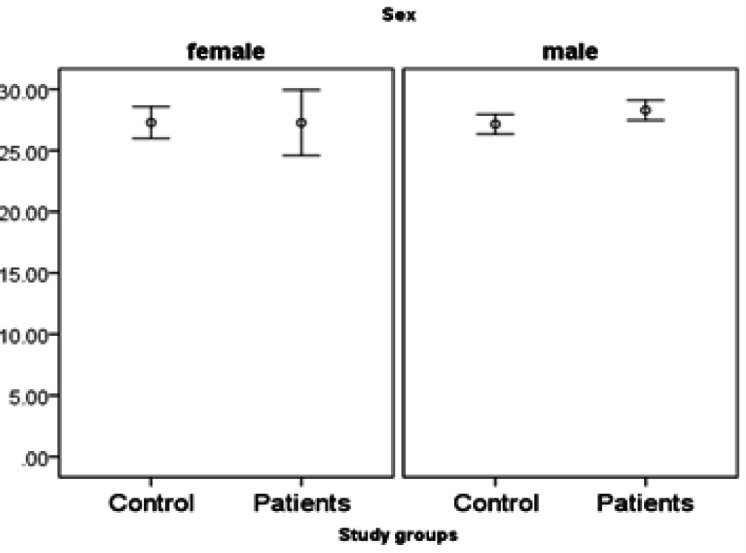
Expression of miR455-5p in Study Groups by Gender

**Figure 3:**
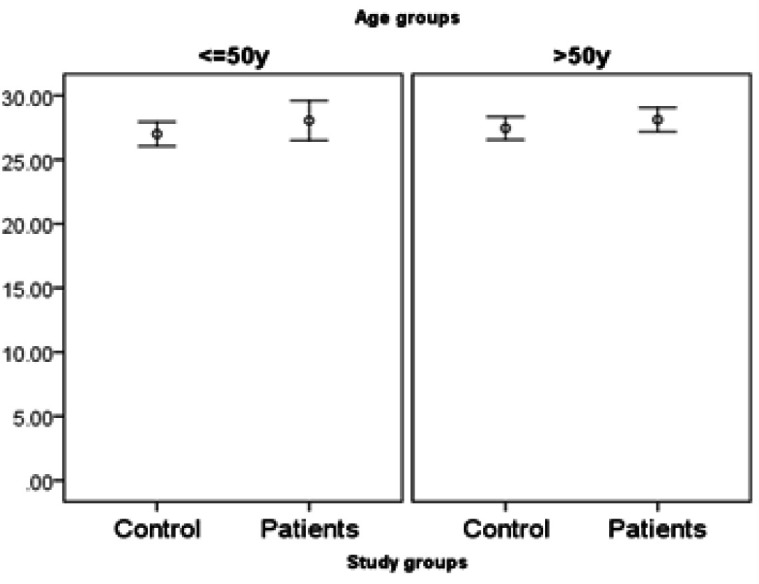
Expression of miR455-5p in Study Groups by Age

*miR455-5p* expression is significantly higher in patients. There is no interaction with age (it means the difference in age under 50 is similar to age over 50). However, the difference of *miR455-5p* is only significant in males but not females (Figures 1, 2 and 3).

**Figure 4:**
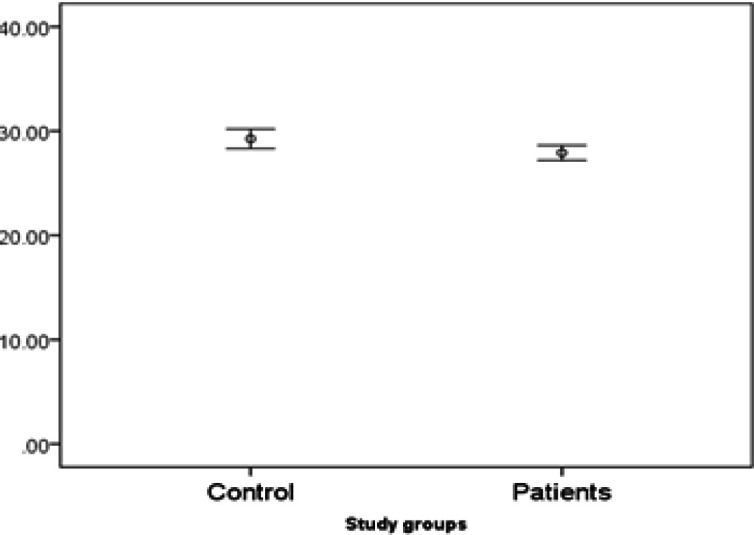
Expression of miR153in Study Groups

**Figure 5:**
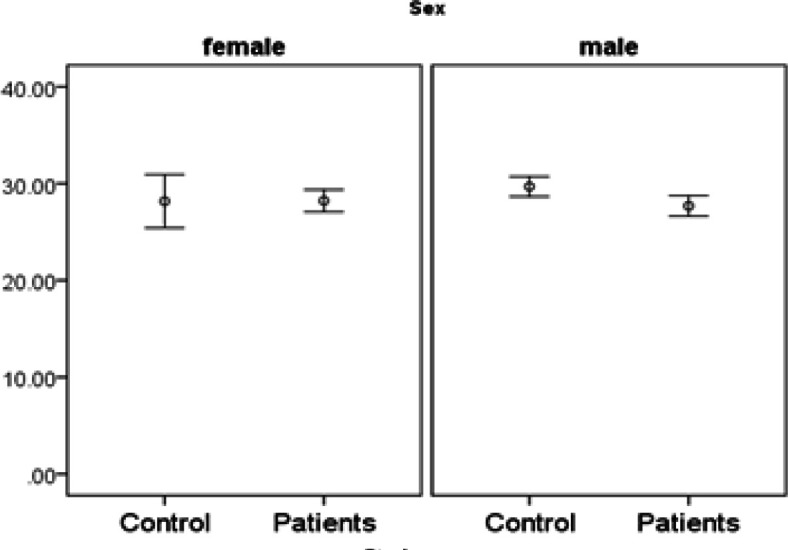
Expression of miR153 in Study Groups by Gender

**Figure 6:**
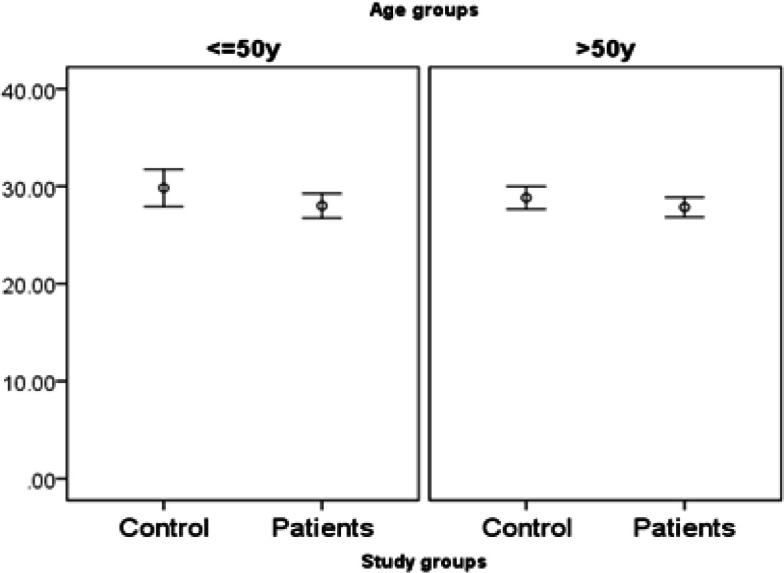
Expression of miR153in Study Groups by Age

*miR153* expression is significantly lower in patients. There are some interactions with both gender and age.In other words, the difference of* miR153* is only significant in males but not females. And the difference is higher in under 50 year-old participants (marginally significant; p-value=0.06)) rather over 50 year-old participants (Figures 4, 5 and 6,)

*Differences in the expression of miRs in two groups*

First,Ct of each sample was determined.The relative differences in expressions were calculatedby Δct method for miR. In this way after performing the calculations:miR 153 expression in patients is relatively1.97 times lower than in healthy individuals.Also *miR 455-5* expression in patients is relatively1.97 times higher than in healthy individuals.

**Table 1:** Demographic Characteristics of Patient and Controls

	Control	Patients
Sex		
Female	7 (28.00%)	10 (40.00%)
Male	18 (72.00%)	15 (60.00%)
Age groups		
<=50y	12 (48.00%)	10 (40.00%)
>50y	13 (52.00%)	15 (60.00%)
Age	50.68±7.41	51.76±6.51

**Figure7:**
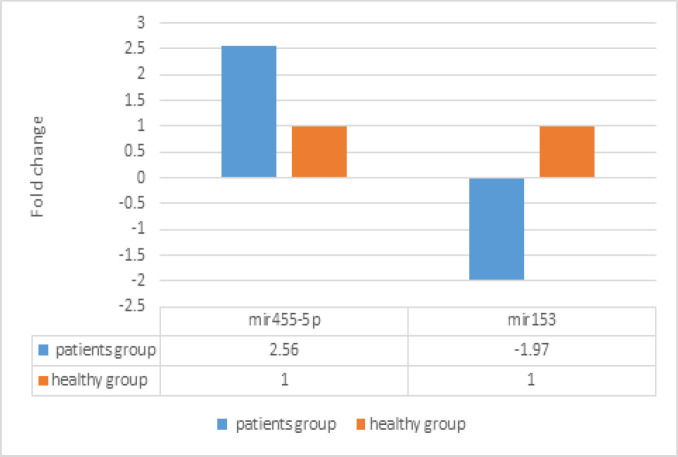
Comparison of Expression Markers of miR153 and miR455-5 in Peripheral Blood of Cancer and Healthy Individuals

## Discussion

Over the past decades, cancer has been identified as a result of genetic and epigenetic changes on tumor suppressor genes and oncogenes. Various regulatory factors typically regulate the expression of these genes, which allows for the proper separation of cell processes, cell differentiation and cell death. In cancer the regulation of these genes, followed by corresponding processes is out of control which ultimately leads to tumor. Recent studies have identified molecular mechanisms and genes involved in tumorigenicity. One of the latest findings is the discovery of miRNA molecules. These molecules are typically involved in various physiological processes and play an important role in the development of cancer.(Friedman et al., 2009)

A large number of miRNAs were studied in relation to OSCC with increased and decreased expression in comparison with healthy subjects including *miR-24*, *miR-31*, *miR-196 a*, *miR-181* typically increase the expression in OSCC. *MiR-223 *and* miR-29 *adecrease the expression in OSCC (Mo et al., 2012; Min et al., 2015).

Jamali et al., (2015) examined the role of. Prognostic value of different microRNAs [miRs] in human head and neck squamous cell carcinoma [HNSCC]. In *miR153*, researchers have found that this miR is tumour suppressor and it has the invasive ability of epithelial cancer cells.

Shan et al., (2015) investigated the role of *miR 153* in human non small cell lung cancer and found that this miR is tumour suppressor. More over there is overexpression of ADAM 19 in relation with *miR 153* expression.

Niu et al., (2015) analyzed the relationship between TGF-β2 and miR153 in Osteosarcoma patients. *miR 153* is tumour suppressor by downregulating *TGF-β2 *expression due to its role in cell proliferation.

Zhang et al., (2015) found the decreased expression levels of miR153 in tissue samples of gastric cancer by suppressing SNAI1 and EMT in the cells of gastric cancer.miR 153 upregulation diminished cell migration and invasion of MKN-45 Cells.Meanwhile *miR153 *downregulation has incentivized cell migration and invasion of SGC-7901. *miR 153* is an important predictive marker for continued existence of gastric cancer patients. 

Ying et al., (2018) examined the role of *miR 455-5p *in esophageal SCC patients. In this study, the factors mentioned on sixty tumor tissues were compared with the adjacent healthy tissue and it was reported that *miR455-5p* can be thought as a miR suppressive tumor and plays a significant role in esophageal SCC by typically suppressing the expression of *Rab31*. It is important to note that soft tissues of the human oral cavity and esophagus are covered everywhere by a stratifying squamous epithelium (Liu et al., 2018).

Early diagnosis is important for better prognosis of oral squamous cell carcinoma.

In conclusion, bio markers like *miR 153* and *miR 455-5p* for OSCC can be worked as a therapeutic intervention.They play a vital role in diagnosing OSCC. It is important to note that the poor OSCC prognosis is primarily due to delayed detection of the disease and lack of appropriate biomarkers to detect tumour growth. Authors recommend that more research is needed in tumour markers to enhance clinical outcome.
